# Arsenic Biotransformation as a Cancer Promoting Factor by Inducing DNA Damage and Disruption of Repair Mechanisms

**DOI:** 10.4061/2011/718974

**Published:** 2011-08-02

**Authors:** Victor D. Martinez, Emily A. Vucic, Marta Adonis, Lionel Gil, Wan L. Lam

**Affiliations:** ^1^Department of Integrative Oncology, BC Cancer Research Centre, 675 West 10th Avenue, Vancouver, BC, Canada V5Z 1L3; ^2^Biomedical Sciences Institute, Faculty of Medicine, University of Chile, Independencia 1027, 8380453 Santiago, Chile

## Abstract

Chronic exposure to arsenic in drinking water poses a major global health concern. Populations exposed to high concentrations of arsenic-contaminated drinking water suffer serious health consequences, including alarming cancer incidence and death rates. Arsenic is biotransformed through sequential addition of methyl groups, acquired from s-adenosylmethionine (SAM). Metabolism of arsenic generates a variety of genotoxic and cytotoxic species, damaging DNA directly and indirectly, through the generation of reactive oxidative species and induction of DNA adducts, strand breaks and cross links, and inhibition of the DNA repair process itself. Since SAM is the methyl group donor used by DNA methyltransferases to maintain normal epigenetic patterns in all human cells, arsenic is also postulated to affect maintenance of normal DNA methylation patterns, chromatin structure, and genomic stability. The biological processes underlying the cancer promoting factors of arsenic metabolism, related to DNA damage and repair, will be discussed here.

## 1. Introduction

Arsenic is one of the most abundant elements in the Earth's crust [1]. Chemically, it is classified as metalloid, exhibiting organic (when linked with carbon and hydrogen) and inorganic (combined with oxygen, chlorine, and sulfur, among other elements) forms [2]. Inorganic arsenic (iAs) can be present naturally in soil, especially in rocks containing copper or lead, and in the atmosphere as airborne dust. Additionally, anthropogenic activities, such smelter operations, can cause water contamination [3, 4]. In the environment, iAs can be found in several oxidation states, more frequently as trivalent (iAs[III], also known as arsenite) and pentavalent (iAs[V] or arsenate) species [5]. These forms are differently metabolized by mammals (see below) and exhibit distinct grades of toxicity.

Several health effects have been documented as a consequence of iAs exposition, with the majority of harmful exposure coming from ingestion through drinking water. iAs-associated malignancies include skin lesions, hypertension, ischemia, some endemic peripheral vascular disorders (e.g., “black foot disease”), severe arteriosclerosis, neuropathies, noticeably, many types of cancer [6–9]. A number of studies have established significant associations and/or dose response trends between iAs in drinking water and occurrence of tumors of the skin, bladder, kidney, liver, prostate, and lungs [10–15]. 

The evidence of a relationship between iAs in drinking water and cancer is extensive and sufficient, leading to the International Agency of Research on Cancer (IARC) to consider this metalloid as a Group 1 human carcinogen. The estimated cancer-death risk associated with daily consumption of 1.6 liters of water with iAs concentrations of 50 *μ*g/L is 21/1,000 [16]. For these reasons, the World Health Organization and the U.S. Environmental Protection Agency have recommended a threshold of 10 *μ*g/L for iAs concentration in drinking water [17, 18]. 

Despite efforts to reduce high-scale exposure, many nations throughout the world have iAs concentrations in water that are above the recommended level [19–21]. Approximately 40 million people worldwide are thought to be exposed to iAs levels that can be considered dangerous [19]. Among them, 21 million people in Bangladesh and India (West Bengal) are exposed to drinking water with i*As* concentrations >50 *μ*g/L [22], and shockingly, iAs concentration in water wells in these areas has been documented as high as 1000 *μ*g/L [23]. In China, it has been estimated that more than 3 million people are exposed to iAs from groundwater [24], while in southwestern Taiwan, some residents have used well water contaminated with iAs for more than 50 years (some ingesting as much as 1000 *μ*g iAs/day) [25–27]. In Northern Chile, the population was exposed to levels of iAs in drinking water around 900 *μ*g/L between 1958 and 1970, with nearby towns registering exposures of 600 *μ*g/mL as late as 1994 [11].

## 2. Arsenic Biotransformation

About 80–90% of ingested As[III] or As[V] is absorbed from the gastrointestinal tract [28–30]. Data derived from autopsies has determined that muscles, bones, kidneys, and lungs have the highest absolute accumulated amounts of iAs, while skin and excretory/storage organs, such as nails and hair, are the most concentrated [31]. iAs[III] exhibits a significantly higher biological activity than As[V]; however, effects observed in mammals could be similar, since absorbed As[V] is mostly reduced to As[III] on the initial steps of arsenic metabolism in mammals [32, 33]. Interestingly, there is evidence for interindividual differences in iAs metabolism/excretion in humans and other species [34, 35].

The biotransformation process of iAs occurs via methylation through alternating reduction of As[V] to As[III], and subsequent addition of methyl groups [36]. This methylation process uses S-adenosylmethionine (SAM) as a methyl group donor, through a SAM-dependant As[III]-methyltransferase, initially isolated from rat liver and a human homologue of cytochrome19 [37]. This enzyme catalyzes the transfer of a methyl group from SAM to As[III] producing methylated and dimethylated arsenic compounds. Trivalent methylated species, such monomethylarsonous acid (MMA[III]) and dimethylarsinic acid (DMA[III]), have been detected in the urine of patients chronically exposed to iAs in drinking water [38, 39]. Methylated pentavalent arsenicals such as monomethylarsonic acid (MMA[V]) and dimethylarsinic acid (DMA[V]) are major metabolites of iAs in human urine, with DMA[V] being the final metabolite in humans [39–41]. Derivate methylated species from iAs metabolisms are considered relevant agents during arsenic carcinogenicity, specially through induction of oxidative stress and impairing DNA repair processes. These aspects will be discussed in the following sections.

Despite evidence of biotransformation role in arsenic carcinogenicity, it has been demonstrated that arsenic can induce malignant transformation in cell lines with deficient arsenic-methylation capacity. Arsenic methylation-deficient RWPE-1 human prostate cells undergo malignant transformation when exposed to 5.0 *μ*M of iAs[III] during 30 weeks [42]. Alternative mechanism of arsenic-induced malignant transformation might be associated with mitochondrial dysfunction (see below), specifically through transcription and replication of the mitochondrial genome, in which the mitochondrial transcription factor A (mtTFA) and its regulators, such the nuclear respiratory factor-1 (NRF-1), play key roles [43, 44]. In this context, it has been demonstrated that mtTFA and NRF-1 expressions levels are increased in cells exposed to iAs[III] in a concentration-dependent manner, suggesting that arsenic regulates mitochondrial activity through an NRF-1-dependent pathway [45].

## 3. Arsenic Carcinogenicity: Role of Oxidative Stress

Despite the strong relationship between iAs exposure and cancer, the exact mechanism is still unknown. There is evidence supporting low level mutagenic activity of iAs; however, it has also been shown that iAs can induce transformation in several cell types [46, 47]. Moreover, iAs can interfere with a number of biological processes, including DNA methylation, since the arsenic biotransformation pathway uses SAM as a methyl group donor. Therefore, epigenetic mechanisms have also been proposed to participate in iAs-induced carcinogenesis [48]. 

Biotransformation of iAs has been proposed to generate final and intermediate metabolites exhibiting higher toxicity and reactivity compared to originally ingested iAs [5, 49, 50]. Methylated species, especially DMA[V], have been demonstrated to be genotoxic and cytotoxic [46, 49, 51–53]. Several studies have shown that DMA[V] can exhibit carcinogenic potential in mammals, mainly in lungs, skin, liver, kidney, thyroid, and urinary bladder [39, 54–58]. It has been proposed that DMA[V] can participate in promoting tumorigenesis of lungs and skin in mouse via the production of dimethylated arsenic peroxide [(CH_3_)_2_AsOO*·*], a type of reactive oxygen species (ROS) generated during iAs metabolism [53, 54].

In the light of these facts, oxidative stress has been proposed as a plausible general mode of action for iAs carcinogenesis [59–63]. Oxidative stress is characterized by generation of several ROS, such as superoxide anion (O_2_^−^), hydroxyl radical (*·*OH), hydrogen peroxide (H_2_O_2_), singlet oxygen (^1^O_2_), and peroxyl radical (LOO), among others [64]. One of the primary species formed in iAs-induced oxidative stress is O_2_^−^, followed by a cascade of secondary ROS such as H_2_O_2_ and *·*OH [61].

iAs exposure results in the generation of ROS in various cellular systems, and its production has been proposed as one of the early biological events on iAs-related carcinogenic process [65]. In addition, cultured vascular endothelial cells exposed to iAs increase oxygen cell consumption contributing to increased ROS production, stimulating cell signaling and activating transcription factors [66]. Conversely, ROS scavengers can suppress arsenic-induced oxidative stress and its cytotoxic effects in cells [67, 68]. It has also been described that iAs exposure can affect expression of genes associated with stress-related components, DNA damage and repair-responsive genes, activation of transcription factors such as the AP-1 complex, and increases in proinflammatory cytokines, which could influence response to acute arsenic toxicity [69]. Alternatively, ROS generation by iAs can involve hepatic and renal heme oxygenase isoform 1, generating among others species, free iron which subsequently participates in reactions where H_2_O_2_ is reduced to OH^−^ and *·*OH [69]. Additionally, the oxidation of iAs[III] to As[V] during formation of intermediary arsine species can also generate H_2_O_2_ [70]. 

Mechanisms of iAs carcinogenicity could vary between different tissues, due to different oxygen concentrations, and accumulation of iAs species, endogenous reducing agents, and ferritin, among others factors [71, 72]. For example, lungs are exposed to the highest oxygen tensions in the body, and DMA[III], and its derivates (including ROS) are excreted through the lung, which could explain why this organ is frequently affected by iAs-induced carcinogens [60].

It has been suggested that arsenic-associated mitochondrial dysfunction, mitochondrial DNA (mtDNA) depletion, and induction of mtDNA deletions may contribute to the carcinogenicity in humans [73]. Also, mitochondria might be an important target of arsenic-induced genotoxicity [74]. On the other hand, since mitochondria is a major source of intracellular ROS, arsenic-mediated disruption of its function can lead to an increase in intracellular ROS levels and subsequently, to an increased mutagenic potential, either directly or by decreasing DNA repair capacity [73]. Relationships between mitochondria and arsenic-mediated effects are supported by observations such as suppression of arsenic-induced apoptosis in HeLa cells by the antioxidant action of N-acetyl-cysteine, which prevents mitochondrial membrane depolarization [75]. Alternatively, arsenic can act directly through condensing mitochondrial matrix and opening of permeability transition pores [76].

## 4. DNA and Chromosomal Damage by iAS-Induced Oxidative Stress

Genotoxic mechanisms associated with arsenic carcinogenicity remain controversial. While some groups argue against this type of interaction, others have postulated this can be a significant mode of action. Rossman [46] has proposed that arsenite does not react directly with DNA. In the same way, toxic doses (10–15 *μ*M) of iAs[III] act as a poor mutagen at the gpt mutagenic target in transgenic Chinese hamster G12 cells [77]. On the other hand, it has been proposed that iAs[III] is a significant mutagen that induces mainly large chromosomal mutations [78]. Alternatively, arsenic has been shown to be mutagenic to mitochondrial DNA and can potentially induce nuclear DNA damage by activating mitochondrial ROS through increased expression of mtTFA [45]. Also, arsenic can induce mutations as well as methylation changes in the mouse testicular Leydig cell genome [79]. Similarly, comet assay performed on human prostate epithelial cells exposed to 100 pg/mL of arsenic exhibited tail-like structures, suggesting induction of nuclear DNA damage [45]. 

iAs is known to damage chromosomes [80]. Due to little evidence of covalent binding between iAs and DNA structures, it has been proposed that much of the DNA damage observed during iAs exposure is indirect, occurring mainly as a result of ROS induction which generates DNA adducts, DNA strand breaks, cross links, and chromosomal aberrations [81, 82]. Figure 2 indicates the sequence of events related to ROS induced DNA damage after iAs exposure. Depending on which cell cycle phase exposure occurs, as a consequence DNA oxidation, iAs can result in gross chromosomal aberrations including DNA strand breaks [61, 69].

## 5. DNA Strand Breaks

**Figure 1:**
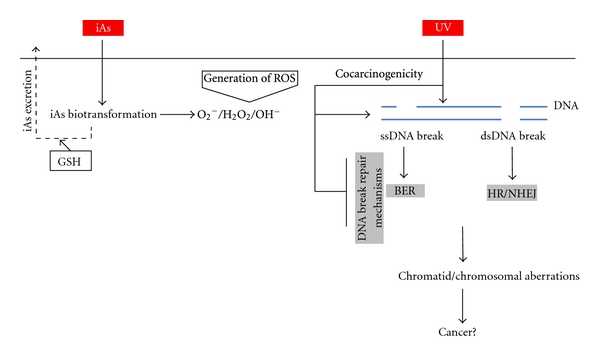
Arsenic-induced DNA strand breaks. After ingestion, iAs biotransformation process could lead to iAs excretion, mainly conjugated with Glutathione (GSH). On the other hand, biotransformation process may generate reactive oxygen species (ROS), probably in a specific sequence: superoxide anions (O_2_^−^), hydrogen peroxide (H_2_O_2_), and hydroxyl radicals (*·*OH). These species can induce both single-strand (ssDNA) and double-strand (dsDNA) breaks by inducing oxidative damage. In parallel, they can inhibit DNA break repair mechanisms both for ssDNA breaks (mainly base excision repair [BER]) and for dsDNA breaks (homologous recombination [HR] and/or nonhomologous end joining [NHEJ]). Additionally, ROS derived from iAs biotransformation can act as cocarcinogens, for example, increasing damage potential of ultraviolet (UV) light. All these events could be associated, in part, to iAs-related carcinogenic mechanism.

**Figure 2:**
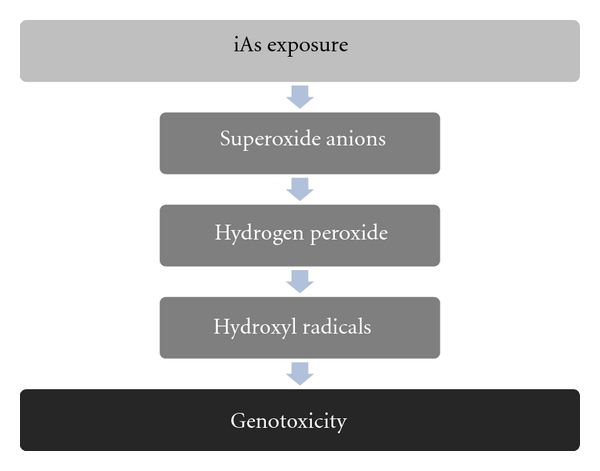
Events related with ROS-induced DNA damage after iAs exposure. Specific sequence of reactive oxygen species generation as a consequence of iAs biotransformation in mammals.

iAs can induce DNA strand breaks even at low concentrations. Main related-events are summarized in Figure 1. Single-strand DNA (ssDNA) breaks are the most common lesions induced by exogenous genotoxins [83]. Arsenic-induced ssDNA breaks are likely caused through ROS, either directly by free-radical attack on the DNA bases or indirectly during the course of base excision repair (BER) mechanisms [84]. Arsenic-induced ROS has been shown to promote ssDNA breaks in mice lungs [70]. Furthermore, human fibroblast cell lines exposed to iAs exhibit ssDNA breaks and DNA-protein adducts, as well as sister chromatid exchanges [85].

iAs is also capable of producing double-strand DNA (dsDNA) breaks at concentrations of 5 *μ*M in mammalian cells [86]. These are one of the most deleterious and mutagenic DNA lesions experienced in human cells, leading to gross losses of genetic material [87]. Therefore, iAs is also proposed to act as a cocarcinogen, exacerbating damage induced by other agents. In this context, 1 *μ*M of iAs increases UVR-mediated DNA strand breaks by interfering with Poly-adenosine diphosphate-ribose polymerase 1 (PAPR-1) activity, which plays an important role in the ssDNA or dsDNA breaks repair process [88].

MMA[III] was found to be a potent clastogen in late G1- or S-phase-treated cells; however, lesions induced by MMA[III] are quickly repaired through BER mechanisms when they are induced in G0- or G1-phase of the cell cycle [84]. Trivalent arsenicals might induce either chromatid- or chromosome-type aberrations during treatment in G0 or G1. If ssDNA or dsDNA breaks produced by iAs-induced ROS pass the S-Phase (DNA synthesis), replication occurs and chromatid- and chromosome-type aberrations can be produced [89]. Evidence pertaining to these type of aberrations is discussed below.

## 6. Arsenic-Induced Chromatid and Chromosomal Aberrations

Arsenic is a known inducer of chromosomal and chromatid aberrations. Lee et al. [90] demonstrated that iAs can effectively induce methotrexate-resistance in mouse 3T6 cells, resulting in selection of cells with amplification of the dihydrofolate reductase gene [91]. Genetic changes were observed in bladder tumor (transitional cell carcinoma, TCC) from 123 patients in Argentina and Chile, exposed to iAs concentrations exceeding 500 *μ*g/L. Individuals exposed to high As concentrations (>300–600 *μ*g/L) exhibited a higher total number of chromosomal aberrations, supporting the hypothesis that exposure to iAs increases genomic instability. Furthermore, chromosomal aberrations (specifically DNA copy-number alterations) were more abundant among iAs-exposed bladder TCC tumors from southwest Taiwan compared with nonexposed tumors from the same area [92]. Some alterations were common to those found in other studies, suggesting that nonrandom events are associated with As-induced urinary TCC formation and progression [93].

Other large-scale cytogenetic aberrations have been observed in iAs-exposed populations. Gonsebatt et al. [94] analyzed cytogenetic effects in individuals exposed to different levels of As in drinking water. People exposed to iAs at an average of 400 *μ*g/L showed a significant increase in frequency of chromatid and isochromatid deletion in first-metaphase lymphocytes and micronuclei in oral and epithelial exfoliated cells compared to individuals with lower exposures. Women and children from the northeast Andean Region of Argentina exposed to 200 *μ*g/L of iAs in drinking water displayed higher micronuclei frequency compared to people exposed to very low iAs concentrations, but did not have altered frequency of other aberrations, such as sister chromatid exchange, specific translocations, or cell-cycle progression [95].

## 7. Oxidative Damage

DNA modifications due to iAs-induced ROS can produce oxidative damage, which can be measured through the presence in urine of products of guanine oxidation in position 8 (8-oxo-2′-deoxyguanosine (8-oxodG), 8-hydroxy-guanine [8-oxo-G], 8-hydroxyguanosine [8-oxy-Guo] and 8-hydroxy-2′-deoxyguanosine [8-OHdG] [64]. After DMA[V] administration in terminal bronchiolar Clara cells from mice, markers for oxidative stress were detected, including 8-oxodG [96]. Additionally, it has been demonstrated that the presence of 8-OHdG was associated with administration of DMA[V] in iAs-related human keratoses, squamous cell carcinoma, basal cell epithelioma, and normal skin from iAs-intoxicated patients [97–99]. Also, iAs[III] can induce 8-OHdG and promote genomic instability by damaging DNA and inducing oncogene expression (including several factors regulating cell cycle progression) human breast cancer MCF-7 adenocarcinoma epithelial cells exposed to iAs[III] [100]. Oral administration of DMA[V] increases 8-oxo-G levels through (CH3)_2_AsOO*·* [54, 55].

## 8. Inhibition of DNA Repair Mechanisms Associated with Arsenic Exposure

iAs can also induce DNA damage by interfering with the DNA repair processes. Inhibition or impairment of the DNA repair processes, principally the repair of DNA strand breaks, is considered one of the main mechanisms of iAs carcinogenesis [88, 101, 102]. For example, DMA[V] affects DNA repair and replication mechanisms in human alveolar cells, leading to persistence DNA damage (mainly apurinic/apyrimidinic sites) and generating ssDNA breaks as a consequence [103, 104]. 

DNA base damage (induced by oxidative stress) can be repaired through excision repair mechanisms, which are subdivided into BER and nucleotide excision repair (NER) pathways [105]. BER is the predominant repair pathway for DNA lesions caused by ROS, and the first candidate in iAs-related DNA repair [69, 106]. Transcription levels of genes related to BER mechanisms are altered in a gene, age-, dose-, and duration-dependent manner in lung tissue of mice exposed to iAs [107]. On the other hand, iAs was also shown to alter BER mechanisms in GM847 lung fibroblasts and HaCaT keratinocytes, increasing levels of BER-related enzymes and repair capacity [108].

Several enzymes participate in the BER mechanism, some of which are known to be modulated by iAs. Among them, DNA polymerase *β* (Pol*β*) and DNA ligase I (LIG1) have been described as affected by As[III] [109, 110]. Normally, after generation of 5′ incision on an abasic site leaving a 3′-hydroxyl and a 5′-deoxyribose 5-phosphate, Pol*β* hydrolyses the 5′-sugar phosphate and adds at least one nucleotide to the 3′-hydroxyl end. The remaining strand is nick sealed by LIG1, and PARP-1 may recruit the required proteins [108]. However, in lung fibroblasts and HaCaT keratinocytes exposed to As[III], Pol *β* mRNA levels are downregulated in a dose-dependent manner (doses >1 *μ*M), and at doses lower than 1 *μ*M both Pol *β* mRNA and protein levels, and consequently, BER activity, were significantly increased [108]. Additionally, this enzyme is stimulated in response to low doses iAs and modulated by other sources of oxidative stress [111–114]. Interestingly DNA copy-number alterations (CNAs) in lung squamous cell carcinoma (SqCC) from iAs-exposed patients from northern Chile contain the Pol*δ* 1 (DNA polymerase *δ* 1, catalytic subunit), which codes for the proofreading domain of the DNA polymerase *δ* complex and also participates in ssDNA breaks repair process [115–119].

It has been proposed that iAs[III] works at transcriptional level to repress a group of genes encoding for DNA repair enzymes participating in BER and NER mechanisms, mainly through its downregulation. This, in combination with other events, contributes to toxicity or cancer [120]. In parallel, changes in expression levels have been also corroborated in human exposed populations. Exposure to arsenic in drinking water was correlated to decreased expression of ERCC1, XPB, and XPF in lymphocytes from exposed individuals [121]. Decreased ERCC1 gene expression was confirmed in lymphocytes treated with > 1 *μ*M of iAs[III], and a significant reduction of ERCC1 protein levels was observed among individuals exposed to drinking water with low levels of arsenic [122]. Similarly, mRNA levels of ERCC1 expression were significantly associated with arsenic concentrations in drinking water, implicating the DNA repair response was induced by arsenic exposure [123]. On the other hand, OGG1 expression (which encodes for 8-oxoguanine DNA glycosylase, involved in base excision repair of 8-oxoguanine [124]) was strongly associated with arsenic concentrations [125], revealing involvement of mechanisms related the effects of arsenic-mediated ROS on DNA.

DNA ligation is a key step in DNA repair pathways [126]. Interestingly, it has been shown that iAs can specifically inhibit this process as well. More recently we have found that the mRNA, protein and activity levels of both DNA ligase I and ligase III are significantly reduced in mammalian cells in response to As[III] [109]. Additionally, As[III] retards DNA break rejoining by interacting with the vicinal dithiols and thus inhibiting DNA ligation [127]. Mammalian cells have been shown to exhibit a dose-dependent decrease in ligase activity with exposure to As[III], corresponding to a decrease in mRNA levels of this enzyme [108, 128]. On the other hand, it has been also shown that LIG1 and other DNA damage/repair genes were increased by As[III] and As[V] treatment, suggesting a cellular response to iAs-induced DNA damage [129].

Members of the poly (ADP-ribose) polymerase (PARP) family also play an important role in the regulation of DNA damage repair. PARP-1 (accounting for about 90% of the total cellular poly ADP-ribose formation) acts as a “DNA damage sensor”, exhibiting high affinity to bind both ssDNA and dsDNA breaks [130–132]. It has been proposed that lack of PARP-1 enhances cellular sensitivity to As[III] [133]. Cells deficient in this gene product display greater telomere attrition. This process can be attributable to susceptibility of the triple-G-containing structures of telomeric DNA to oxidative damage [134, 135]. In parallel, cells deficient in PARP-1 exhibit reduced repair of 8-oxoguanine, another marker for oxidative damage that can potentially be induced by iAs [136]. Finally, specific CNAs located at located at 10q11.23 in lung SqCC from iAs-exposed patients from northern Chile contain the PARG (polyADP-ribose glycohydrolase) gene, which also participates in ssDNA breaks repair process [102, 115, 137].

## 9. Genomic Landscape of Arsenic-Related Lung Cancer

Lungs are the most frequently affected organ by iAs, and lung cancer remains the main cause of iAs-related death [24]. Tobacco exposure is the main aetiological factor in lung cancer; however, iAs ingestion through drinking water also represents a risk factor, particularly for lung squamous cell carcinomas (SqCCs). Interestingly, the incidence of SqCC is decreasing worldwide and is usually associated with cigarette smoking, but in Northern Chilean populations exposed to arsenic contaminated drinking water, SqCC frequently occurs in never smokers, [14, 138] suggesting distinct molecular tumorigenic pathways may underlie arsenic-related cancers. 

**Figure 3:**
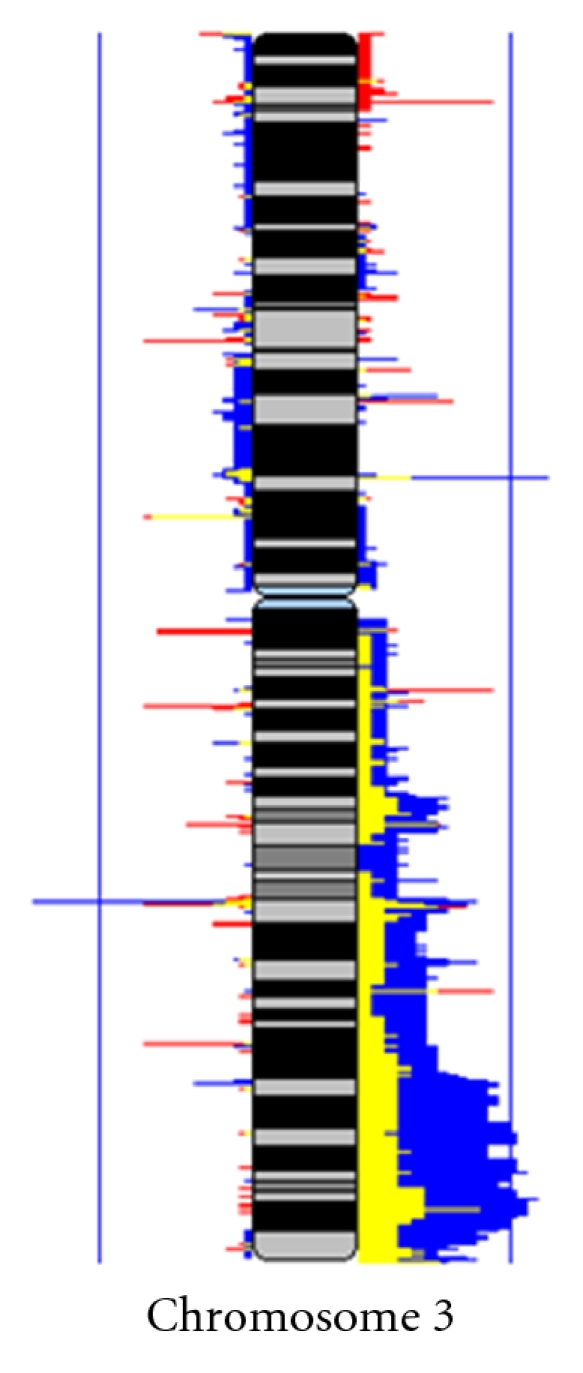
Comparison of CNA frequency at chromosome 3 between lung SqCC exposed and nonexposed to iAs. The figure represents a comparison of CNA frequency at chromosome 3 generated from 52 lung SqCC biopsies by using a submegabase resolution tiling-set rearray (SMRTr) platform. Of those, 22 derived from arsenic-exposed smokers and never smokers patients from Northern Chile (red) and 30 were current and ex-smokers North American patients without known arsenic exposure nonexposed (blue). Frequency of alteration results for exposed and nonexposed SqCCs cases has been overlaid in this figure, with regions in yellow, denoting a sector of overlapping alteration status in both groups. The magnitude of red, yellow and blue bars represents percentage of samples exhibiting corresponding alteration (0–100%, with blue vertical lines representing 50% frequency). DNA gains and losses are represented to the right and left of chromosome, respectively. Adapted from Martinez et al. [115].

To this effect, it was determined if globally, there existed CNAs specific to lung SqCC cases from a Northern Chilean population chronically exposed to iAs in drinking water [115], using a whole genome tiling-path array comparative genomic hybridization (CGH) platform [139]. It was detected a surprisingly low frequency of DNA gains at chromosome arm 3q in lung SqCCs from arsenic-exposed individuals (Figure 3), which is remarkable, since DNA gains at 3q are the most widely reported alteration associated with lung SqCC tumors and cell lines [140, 141].

It was also identified specific DNA gains and losses associated with lung SqCC from never smokers exposed to iAs. For example, a specific and frequent DNA gain at 19q13.33 contains genes related to ssDNA breaks repair process (POLD1) and neoplastic processes (SPIB and NR1H2). Additionally, a widespread association of DNA copy number loss specific to iAs-exposed lung SqCC, concordant with previous findings showing that arsenic can induce multiple large deletions through the creation of ROS [142] was identified. Some of these deletions, mainly at 9q12, may be relevant to iAs carcinogenic mechanisms, since they have been described in other iAs-related types of cancer and involve genes from the forkhead box (Fox) gene family, which have been linked to tumorigenesis and cancer progression [143].

This recent information provides evidence of distinct CNAs associated with lung SqCC occurring in patients who had exposure to iAs in drinking water and suggests that alternative molecular pathways are activated in this disease subset.

## 10. Conclusion

Arsenic exposure through contaminated drinking water poses a major health concern for over 40 million people worldwide, where for some, arsenic levels are almost 10 times higher than recommended thresholds. In addition to causing a variety of health problems including vascular and neurological conditions, arsenic is an established carcinogen. The rate of cancer incidence and mortality in populations exposed to arsenic contaminated drinking water is alarming. These populations experience particularly exacerbated rates of cancer in organs where arsenic is most concentrated or is excreted, including lung, bladder, and skin cancers. The mechanisms of arsenic-induced carcinogenesis are slowly being elucidated through the study of the precise DNA damaging and cytotoxic properties related to the biotransformation, metabolism, and excretion of arsenic. Discovery of particular genomic and epigenomic lesions induced by this metalloid should encourage a comprehensive approach to elucidate how arsenic can induce different types of cancer. Despite histology similarity, the possibility of iAs-induced cases biologically distinct entities, compared to those induced by other environmental carcinogens, must be considered. Knowledge related to these processes may lead to specific treatment strategies targeting arsenic-induced disorders and malignancies.
